# Trimodal Race Model Inequalities in Multisensory Integration: I. Basics

**DOI:** 10.3389/fpsyg.2017.01141

**Published:** 2017-07-11

**Authors:** Hans Colonius, Felix Hermann Wolff, Adele Diederich

**Affiliations:** ^1^Cognitive Psychology Lab, Department of Psychology, University of Oldenburg Oldenburg, Germany; ^2^Cluster of Excellence ‘Hearing4All,’ University of Oldenburg Oldenburg, Germany; ^3^Cognitive Science Lab, Life Sciences and Chemistry, Jacobs University Bremen Bremen, Germany

**Keywords:** multisensory integration, race model inequality, context invariance, trimodal case, redundant signals effect, superposition model, statistical facilitation, probability summation

## Abstract

The race model inequality has become an important testing tool for the analysis of redundant signals tasks. In crossmodal reaction time experiments, the strength of violation of the inequality is taken as measure of multisensory integration occurring beyond probability summation. Here we extend previous results on trimodal race model inequalities and specify the underlying context invariance assumptions required for their validity. Some simulation results comparing the race model and the superposition model for Erlang distributed random variables illustrate the trimodal inequalities.

## 1. Introduction

When stimulus information, perceived via several sensory modalities, indicates the occurrence of some event, an observer is faster detecting and responding to the stimulus compared to receiving only unimodal information, given a background of noisy signals. As a daily-life example consider the warning lights and siren of an ambulance in a traffic environment, a common audio-visual signal that allows, e.g., a driver to initiate an adequate reaction like giving way faster than if only acoustic or only visual information was available. Since the pioneering study by Todd (1912), this *redundant signals effect* (RSE) has frequently been replicated under laboratory conditions for crossmodal redundant signals combining different modalities, for both manual and saccadic reaction times (RT), and under different experimental conditions (e.g., divided vs. focused attention) (e.g., Miller, 1982; Gielen et al., 1983; Diederich and Colonius, 1987; Corneil et al., 2002).

A number of different models for the mechanisms underlying the RSE have been suggested. Raab (1962) proposed that race models could explain the speedup of responses. Race models assume that (a) each individual stimulus elicits a modality-specific process performed in parallel with the others, and (b) the winners time determines the observable RT, which will also consist of other components like motor execution time. This model implies that the RSE is generated by *statistical facilitation*, or *probability summation*: If latencies are interpreted as (non-negative) random variables, the time to respond to the first of several redundant signals is faster, on average, than the response time to a single signal. More generally, Miller (1982) observed that for the race model with stimuli *x* and *y*, the following inequality should hold:

(1) $$\begin{array}{l} F_{xy}\left(t\right)\leq F_{x}\left(t\right)+F_{y}\left(t\right), \end{array}$$

for all non-negative time points *t*, with *F*_*xy*_, *F*_*x*_, and *F*_*y*_ denoting the distribution function for the redundant-signals condition and the single-stimulus conditions, respectively. Literature on this race model inequality (RMI) test involving different sensory modalities is huge (for a recent review, see Gondan and Minakata, 2016), likely due to the following reason: a statistically significant violation of the inequality for some value of *t* suggests that the observed speedup of the response cannot be accounted for entirely by probability summation and some, additional or alternative, *coactivation* mechanism has to be postulated.

Numerous modeling approaches for a coactivation mechanism have been proposed (Diederich, 1995) (see also Diederich and Colonius, 2012, for an overview). In contrast to the race model, to our knowledge they all are based on assuming some specific probability distribution or stochastic process. The one adopted for the simulations described below is introduced in Section 3.

Sometimes, instead of inequality (1), the race model is tested using inequality

(2) $$\begin{array}{l} F_{xy}\left(t\right)\leq F_{x}\left(t\right)+F_{y}\left(t\right)-F_{x}\left(t\right)F_{y}\left(t\right). \end{array}$$

However, this is not generally recommended since it is more restrictive than (1) by assuming stochastic independence between the random latencies. While this assumption is not required by the general race model, there is another, essential assumption hidden in any version of the model, known as “context independence” or “context invariance”: the processing of a stimulus of a given modality does not depend on which and how many stimuli from other modalities are presented concurrently (e.g., Luce, 1986, pp. 128–129; Colonius, 1990; Colonius and Vorberg, 1994; Townsend and Wenger, 2004; Gondan and Minakata, 2016). For a more general discussion using coupling theory, see Colonius (2016). A formal definition of this assumption is presented in the next section.

In the majority of RT studies on the RSE, only the bimodal case has been tested. Exceptions are, without claiming exhaustiveness, (Diederich, 1992a, 1995; Diederich and Colonius, 2004; Hecht et al., 2008; Oskarsson et al., 2012; Wang et al., 2012, 2013; Pomper et al., 2014; Hagmann and Russo, 2016), but it seems that no systematic investigation of all possible bimodal and trimodal RMIs and their interdependencies has been performed so far. Here we first specify the context invariance assumptions underlying the inequalities and then discuss various types of trimodal RMIs that can be tested assuming different patterns of (non-) violation of the inequalities. This is illustrated with a first set of simulations comparing the race model with a coactivation model.

## 2. Generalized race model inequalities

In most cases, the stimuli being tested for multisensory integration are from the visual, auditory, or somatosensory modality. Many notable studies also involve other modalities (e.g., Gu et al., 2008; Hoechenberger et al., 2015; Kaliuzhna et al., 2016), like vestibular and olfactory stimuli but, for simplicity, we refer to the first three here only. We write V for the unimodal visual, AV for the bimodal visual-auditory, and AVS for the trimodal visual-auditory-somatosensory condition, with the remaining obvious uni- and bimodal cases denoted accordingly.

### 2.1. The “context invariance” assumption of the race model

Let *A, V, S* be random latencies corresponding to stimulus modalities A,V, and S, respectively. *F*_*A*_, *F*_*V*_, and *F*_*S*_, denote the distribution function for the unimodal conditions A,V, and S respectively. For bimodal condition AV, *H*_*AV*_ is the bivariate distribution function of random vector (*A, V*), and for trimodal condition AVS, *H*_*AVS*_ stands for the trivariate distribution function of (*A, V, S*). Thus, for example,

$$\begin{array}{l} H_{AVS}\left(s,t,u\right)=P\left(A\leq s,V\leq t,S\leq u\right), \end{array}$$

for all *s, t, u* ≥ 0. Moreover, for marginal distributions we write, e.g.,

$$\begin{array}{l} H_{AV}\left(s,\infty \right)\text{ for }P\;\left(A\leq s,V<\infty \right),\text{etc}. \end{array}$$

The formal definition of “context invariance” is as follows:

**DEFINITION 1**. For all *s, t, u* ≥ 0, necessary and sufficient conditions for (trimodal) complete context invariance are

$$\begin{array}{l} H_{A}\left(s\right)=H_{AV}\left(s,\infty \right)=H_{AS}\left(s,\infty \right)=H_{AVS}\left(s,\infty ,\infty \right)\\ H_{V}\left(t\right)=H_{AV}\left(\infty ,t\right)=H_{VS}\left(t,\infty \right)=H_{AVS}\left(\infty ,t,\infty \right)\\ H_{S}\left(u\right)=H_{VS}\left(\infty ,u\right)=H_{AS}\left(\infty ,u\right)=H_{AVS}\left(\infty ,\infty ,u\right) \end{array}$$

and

$$\begin{array}{l} H_{AV}\left(s,t\right)=H_{AVS}\left(s,t,\infty \right)\\ H_{VS}\left(t,u\right)=H_{AVS}\left(\infty ,t,u\right)\\ H_{AS}\left(s,u\right)=H_{AVS}\left(s,\infty ,u\right). \end{array}$$

In other words, complete context invariance holds in the trimodal case if the distributions in the unimodal and bimodal conditions are identical to the corresponding univariate and bivariate marginal distributions of the trivariate distribution. As recently argued in Miller (2016), context invariance is an essential part of the race model concept.

### 2.2. Proving race model inequalities

The proof of Inequality 1 relies on a simple probability inequality. Rewrite (1) for the AV condition as

$$\begin{array}{l} F_{AV}\left(t\right)\leq F_{A}\left(t\right)+F_{V}\left(t\right). \end{array}$$

For any *t* ≥ 0, we define events

$$B_{t}=\left\{A\leq t\right\}\text{ and }C_{t}=\left\{V\leq t\right\}.$$

Because of context invariance, *F*_*AV*_(*t*) = *P*(*B*_*t*_ ∪ *C*_*t*_), and the inequality follows from

$$\begin{array}{l} P\left(B_{t}\cup C_{t}\right)=P\left(B_{t}\right)+P\left(C_{t}\right)-P\left(B_{t}\cap C_{t}\right)\\ \leq P\left(B_{t}\right)+P\left(C_{t}\right), \end{array}$$

where the last probability inequality is known as a special case of “Boole's inequality” (e.g., Diederich, 1992b). It is important to recognize the role of the context invariance assumption here: it guarantees that events *B*_*t*_ and *C*_*t*_ are defined on the same probability space for all *t* or, more generally, that there exists a bivariate distribution *H*_*AV*_(*s, t*) with marginals equal to *F*_*A*_(*s*) and *F*_*V*_(*t*), respectively: *H*_*AV*_(*s*, ∞) = *F*_*A*_(*s*) and *H*_*AV*_(∞, *t*) = *F*_*V*_(*t*).

### 2.3. Generalized race model inequalities: complete context invariance

In the following, we assume that trimodal context invariance holds unless indicated otherwise. In order to avoid trivial upper bounds larger than 1, the right-hand side of all inequalities presented may be replaced by, e.g., min{*F*_*A*_(*t*) + *F*_*V*_(*t*), 1}, etc. For simplicity, we do not state this explicitly.

For further reference, let us start with a listing of all possible bimodal RMIs:

(3) $$\begin{array}{l} F_{AV}\left(t\right)\leq F_{A}\left(t\right)+F_{V}\left(t\right), \end{array}$$

(4) $$\begin{array}{l} F_{VS}\left(t\right)\leq F_{V}\left(t\right)+F_{S}\left(t\right), \end{array}$$

(5) $$\begin{array}{l} F_{AS}\left(t\right)\leq F_{A}\left(t\right)+F_{S}\left(t\right). \end{array}$$

A straightforward generalization to the trimodal case is

(6) $$\begin{array}{l} F_{AVS}\left(t\right)\leq F_{A}\left(t\right)+F_{V}\left(t\right)+F_{S}\left(t\right), \end{array}$$

which follows again as special case of Boole's inequality.

As shown in Diederich (1992b), Bonferroni-type probability inequalities (Worsley, 1982) can be used to derive further trimodal RMIs:

(7) $$\begin{array}{l} F_{AVS}\left(t\right)\leq F_{AV}\left(t\right)+F_{AS}\left(t\right)-F_{A}\left(t\right), \end{array}$$

(8) $$\begin{array}{l} F_{AVS}\left(t\right)\leq F_{VS}\left(t\right)+F_{AS}\left(t\right)-F_{S}\left(t\right), \end{array}$$

(9) $$\begin{array}{l} F_{AVS}\left(t\right)\leq F_{AV}\left(t\right)+F_{VS}\left(t\right)-F_{V}\left(t\right). \end{array}$$

A sharper^1^ bound for *F*_*AVS*_(*t*) results by taking the minimum (at each value of *t*) across all three bounds in 7–9:

(10) $$\begin{array}{l} F_{AVS}(t)\leq \min\{F_{AV}(t)+F_{AS}(t)-F_{A}(t),F_{VS}(t)+F_{AS}(t)\\ \;-F_{S}(t),F_{AV}(t)+F_{VS}(t)-F_{V}(t)\}. \end{array}$$

Given the bimodal inequalities 3–5 hold, the trimodal inequalities 7–9 are sharper than Inequality 6. For example, if Inequalities 3 and 4 are satisfied, Inequality 9 implies Inequality 6:

$$\begin{array}{l} F_{AVS}\left(t\right)\leq F_{AV}\left(t\right)+F_{VS}\left(t\right)-F_{V}\left(t\right)\\ \leq F_{A}\left(t\right)+F_{V}\left(t\right)+F_{V}\left(t\right)+F_{S}\left(t\right)-F_{V}\left(t\right)\\ =F_{A}\left(t\right)+F_{V}\left(t\right)+F_{S}\left(t\right). \end{array}$$

### 2.4. Generalized race model inequalities with restricted context invariance

Next we consider a situation where, for example, data from conditions AVS, AV, and S are available but none from V or A. Responses under the bimodal condition AV can then be conceived as representing reaction times to a “combined” visual-auditory modality formally equivalent to a unimodal condition. The underlying distribution function is bivariate,

$$\begin{array}{l} H_{\left(AV\right)S}\left(w,z\right)=P\left(AV\leq w,S\leq z\right), \end{array}$$

for all *w, z* ≥ 0, with *AV* denoting the RT in condition AV. Context invariance is restricted to the bivariate case, that is:

(11) $$\begin{array}{ll} H_{\left(AV\right)S}\left(w,\infty \right) & =H_{AV}\left(w\right), \end{array}$$

(12) $$\begin{array}{ll} H_{\left(AV\right)S}\left(\infty ,z\right) & =H_{S}\left(z\right) \end{array}$$

The race model then implies

(13) $$\begin{array}{l} F_{AVS}\left(t\right)\leq F_{AV}\left(t\right)+F_{S}\left(t\right). \end{array}$$

Obviously, with adding the following two, there are three inequalities in total:

(14) $$\begin{array}{ll} F_{AVS}\left(t\right) & \leq F_{VS}\left(t\right)+F_{A}\left(t\right), \end{array}$$

(15) $$\begin{array}{ll} F_{AVS}\left(t\right) & \leq F_{AS}\left(t\right)+F_{V}\left(t\right), \end{array}$$

with the corresponding, mutually incompatible, restricted context invariance assumptions.

An alternative situation for considering Inequalities 13–15 is when all three univariate distributions are available but violations occur for some of the univariate pairs. For example, there may be one or more values *t*′ such that

$$\begin{array}{l} F_{AV}\left(t^{\prime }\right)>F_{A}\left(t^{\prime }\right)+F_{V}\left(t^{\prime }\right). \end{array}$$

While the race model for condition AV would be ruled out in this case, the joint processing of *AV* and *S* may still be consistent with a race.

## 3. An illustration with simulated data sets

In this section, we (i) illustrate a possible simulation approach and (ii) point to a specific aspect of dependency occurring for trimodal race models.

### 3.1. Erlang distribution simulation

Simulating the race model and comparing it with a coactivation model requires specifying some RT distributions. Here we select distribution functions derived from the most basic stochastic counting process, i.e., the *Poisson process*. The time between two randomly occurring events follows an exponential distribution with intensity rate λ. Stimulus processing time is defined as the waiting time of the Poisson process for *c*-th event. Empirically, criterion *c* may be influenced by the experimental condition. For example, rewarding high detection accuracy would increase the threshold (Luce, 1986) and higher values of *c* will result in longer detection time, denoted *D*. The distribution of *D* is known as *Erlang* distribution, a special case of the *gamma* distribution with an integer-valued shape parameter *c* and rate λ:

$$\begin{array}{l} D\sim \text{Gamma}\left(c,\lambda \right). \end{array}$$

A rate parameter λ^*x*^, *x* ∈ {*A, V, S*}, has to be specified for each single stimulus condition, auditory (A), visual (V), and somato-sensory (S), respectively, while threshold parameter *c* is assumed to be constant across the modalities. For simplicity, we neglect residual processes, like motor time, and assume that *D* equals the observed reaction time.

For *x, y, z* pairwise different modalities chosen from {*A, V, S*}, detection time in the race model for trimodal redundant stimulation, *D*_*xyz*_, or *D*_*xy*_ for bimodal stimulation, is defined as the minimum of the corresponding single stimulus detection times *D*_*x*_, *D*_*y*_, *D*_*z*_ :

$$D_{xyz}=\min\left(D_{x},D_{y},D_{z}\right)\text{ or }D_{xy}=\min\left(D_{x},D_{y}\right)$$

For a coactivation model, we choose the *superposition model* proposed in Schwarz (1989) (see also Diederich and Colonius, 1991). While a number of alternative coactivation models are available, choosing one based on the Poisson process has the advantage that, with one and the same set of values for the parameters, detection time *D* can be simulated either for the race model or for the superposition model. For the latter, detection time for redundant stimuli follows again an Erlang/Gamma distribution with the intensity rate given by the *sum* of the single stimulus intensity rates λ_*x*_, λ_*y*_, λ_*z*_:

$$\begin{array}{l} D_{xyz}\sim \text{Gamma}\left(c,\lambda_{x}+\lambda_{y}+\lambda_{z}\right)\text{ or }D_{xy}\sim \text{Gamma}\left(c,\lambda_{x}+\lambda_{y}\right). \end{array}$$

The prediction of the race model, an exponential distribution of RTs, is of course not consistent with typical data. It is taken here just for illustration; for fitting empirical data, it would be easy to add a Gaussian component, resulting in an ex-Gaussian distribution. For an empirical evaluation of race and superposition models we refer to Diederich (1992a).

**Figure 1:**
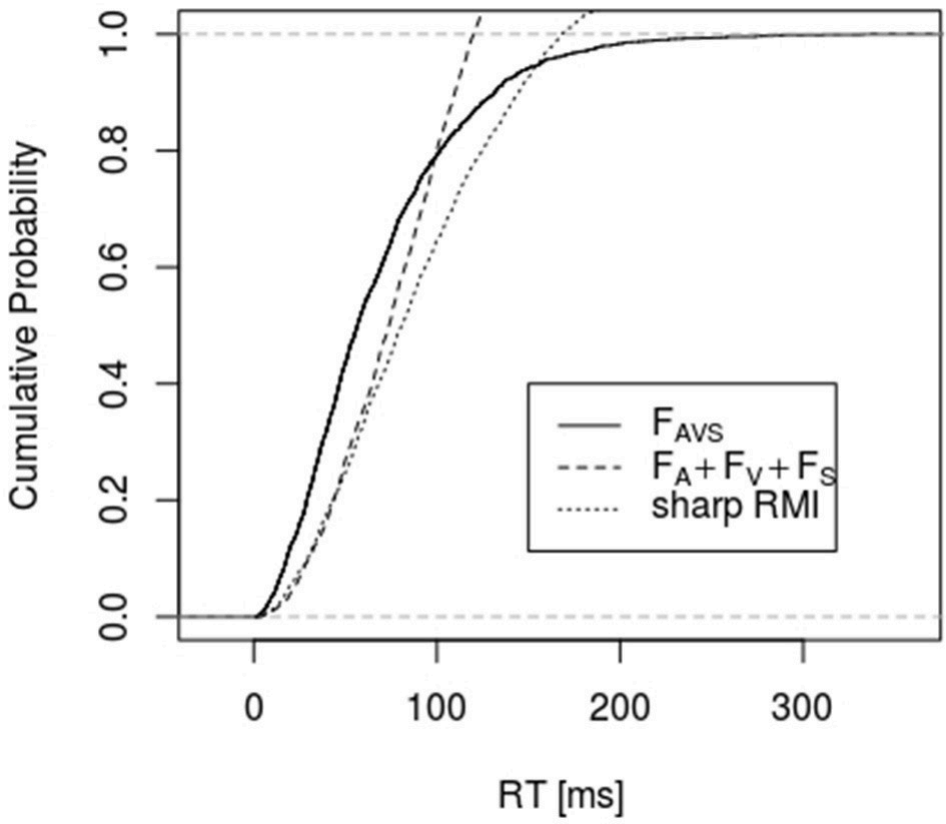
It shows the empirical distribution function of the simulated data for condition AVS and the bounds described by Inequality 6 and 10 (denoted as “sharp RMI”). RTs for condition AVS were simulated according to the superposition model, whereas RTs for all other conditions were generated according to the race model (*n* = 2,000, *c* = 2, λ_*A*_ = λ_*V*_ = λ_*S*_ = 0.01).

**Figure 2:**
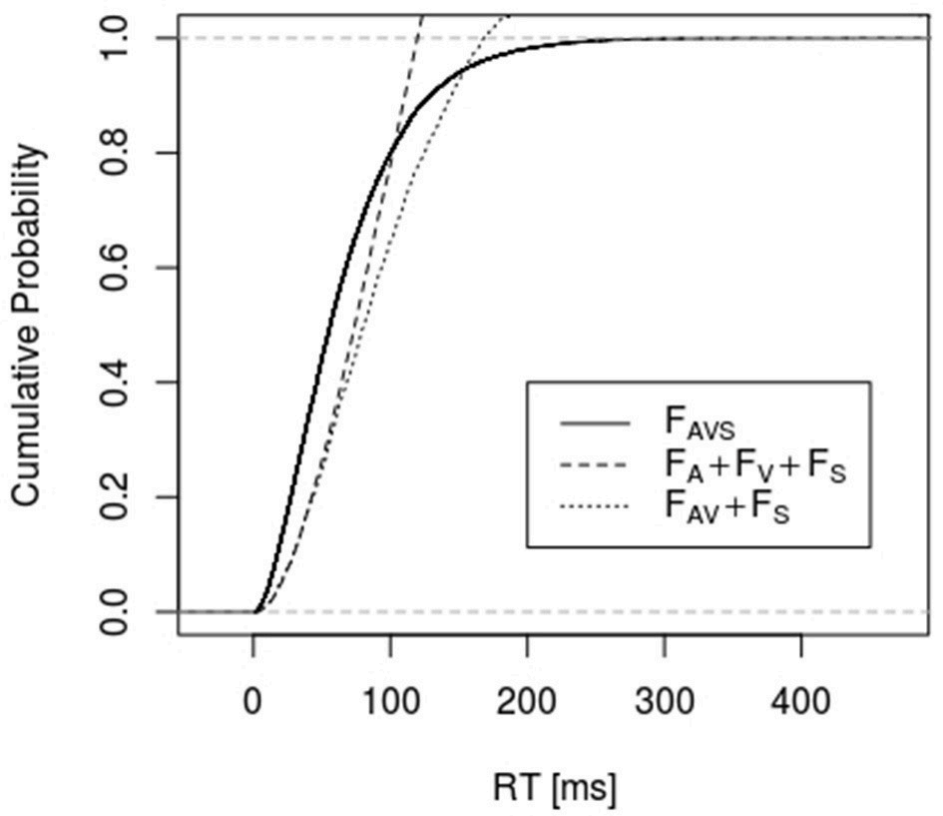
It shows the empirical distribution function of the simulated data for condition AVS and the bounds described by Inequalities 6 and 13. RTs for condition AVS were simulated according to a superposition model, whereas RTs for condition AV were generated according to the race model (*n* = 2,000, *c* = 2, λ_*A*_ = λ_*V*_ = λ_*S*_ = 0.01).

Figures 1, 2 depict empirical distribution functions obtained from simulations of race and superposition models with parameter values *c* = 2, λ_*A*_ = λ_*V*_ = λ_*S*_ = 0.01 with sample size *n* = 2,000. In both figures the trimodal distribution was obtained from the superposition model, i.e., a Gamma (2, 0.03) distribution. Figure 1 compares it to the bounds described by Inequality 6 and 10 (denoted as “sharp RMI”). Both bounds are violated for a large range of percentiles, with the latter bound being violated even for percentiles beyond 80%. Figure 2 compares the trimodal distribution of the superposition model with data from simulating the bounds described by Inequalities 6 and 13 for the race model. Again, there are large-range violations of the bounds. *F*_*AV*_ in bound 13 was obtained from the race model but, in principle, it could be made arbitrarily close to 1 by choosing some coactivation model for condition AV.

### 3.2. Trimodal dependency: adjusting correlations

One characteristic of the race model is the possibility to increase the size of the redundant signals effect by tweaking the correlations between the involved detection processes. Given a race between two detection processes, consider their random processing times, *X* and *Y*, varying from trial to trial. By definition, the shortest processing time min(*X, Y*) determines the detection time for a trial. Negative correlation means that, when one process is fast, the other tends to be slow in a given trial. Thus, there would only be few trials where both processes are rather slow. This results in decreased average detection times, since long times for one process are replaced by shorter times of the other process. One can show that a race model with two processes and maximal negative correlation yields the maximum redundant signals effect (Miller, 1982; Colonius, 1990, 2016).

However, a situation with three “competing” processes *X, Y, Z*, e.g., audio, visual, and somato-sensory, is more complicated. First, instead of one there are three correlation coefficients *r*_*xy*_, *r*_*xz*_, *r*_*yz*_. For a fixed set of two-sample Neyman–Pearson correlation coefficients *r*_*xy*_ and *r*_*xz*_, the third coefficient *r*_*yz*_ can not vary freely between −1 and +1 but is restricted to a narrower range (Stanley and Wang, 1969):

$$\begin{array}{l} r_{xy}r_{xz}-\sqrt{\left(r_{xy}^{2}-1\right)\left(r_{xz}^{2}-1\right)}\leq r_{yz}\leq \sqrt{\left(r_{xy}^{2}-1\right)\left(r_{xz}^{2}-1\right)}\\ +\;r_{xy}r_{xz}. \end{array}$$

Second, values for the correlations generating maximal facilitation are not as trivial to find as in a two-process situation. Limited by the above mentioned constraints, it is not possible to construct a correlation matrix with coefficients *r*_*xy*_ = *r*_*xz*_ = *r*_*yz*_ = −1. Our simulations with a multivariate gamma distribution (not presented here) suggest, for example, that setting *r*_*xy*_ = *r*_*xz*_ = *r*_*yz*_ = −0.5 yields a relatively large redundant signals effect when processing times *X*, *Y*, *Z* have an identical underlying distribution.

## Conclusion and outlook

We have shown that the race model inequality extends naturally from the bimodal to the trimodal case, as long as essential assumptions about context invariance are specified. Moreover, the trimodal case permits “mixed models,” that is, models (i) where the race assumption is only valid for certain modality combinations but not for others, and (ii) where not all unimodal distributions may be available.

For an application of the generalized race model inequalities presented here, the next step is to extend the current statistical tests developed for the bimodal case to the different trimodal cases (for a recent overview, see Gondan and Minakata, 2016). This will also require extensive simulation work as in Kiesel et al. (2007) including an extension to introduce intersubject variability. Finally, it is well-known that the upper bound of the bimodal race model inequality corresponds to maximal negative dependency between the two processes (Miller, 1982; Colonius, 1990, 2016); a particularly challenging task for future study is to characterize the new race model inequalities with respect to the trivariate statistical dependencies underlying their bounds.

## Author contributions

HC, FW, and AD conceived of the analysis. FW performed the simulations. HC and FW wrote the paper.

### Conflict of interest statement

The authors declare that the research was conducted in the absence of any commercial or financial relationships that could be construed as a potential conflict of interest.

## Footnotes

^1^By “sharper” we mean “strictly smaller or equal.”
